# Feasibility of Functional Near-Infrared Spectroscopy (fNIRS) to Investigate the Mirror Neuron System: An Experimental Study in a Real-Life Situation

**DOI:** 10.3389/fnhum.2018.00086

**Published:** 2018-03-05

**Authors:** Pei-Pei Sun, Fu-Lun Tan, Zong Zhang, Yi-Han Jiang, Yang Zhao, Chao-Zhe Zhu

**Affiliations:** ^1^State Key Laboratory of Cognitive Neuroscience and Learning, Beijing Normal University, Beijing, China; ^2^IDG/McGovern Institute for Brain Research, Beijing Normal University, Beijing, China; ^3^Center for Collaboration and Innovation in Brain and Learning Sciences, Beijing Normal University, Beijing, China

**Keywords:** social interaction, mirror neuron system, fNIRS, real-life situation, double density, channel-based group analysis, ROI-based group analysis

## Abstract

The mirror neuron system (MNS), mainly including the premotor cortex (PMC), inferior frontal gyrus (IFG), superior parietal lobule (SPL), and rostral inferior parietal lobule (IPL), has attracted extensive attention as a possible neural mechanism of social interaction. Owing to high ecological validity, functional near-infrared spectroscopy (fNIRS) has become an ideal approach for exploring the MNS. Unfortunately, for the feasibility of fNIRS to detect the MNS, none of the four dominant regions were found in previous studies, implying a very limited capacity of fNIRS to investigate the MNS. Here, we adopted an experimental paradigm in a real-life situation to evaluate whether the MNS activity, including four dominant regions, can be detected by using fNIRS. Specifically, 30 right-handed subjects were asked to complete a table-setting task that included action execution and action observation. A double density probe configuration covered the four regions of the MNS in the left hemisphere. We used a traditional channel-based group analysis and also a ROI-based group analysis to find which regions are activated during both action execution and action observation. The results showed that the IFG, adjacent PMC, SPL, and IPL were involved in both conditions, indicating the feasibility of fNIRS to detect the MNS. Our findings provide a foundation for future research to explore the functional role of the MNS in social interaction and various disorders using fNIRS.

## Introduction

Humans are highly social animals and social interaction is ubiquitous and essential for our survival (Oberman et al., 2006; Sänger et al., 2011; Babiloni and Astolfi, 2014). Understanding the complex social interaction process and its neural basis is the continual focus of social cognitive neuroscience.

Rizzolatti et al. (1996a) first discovered mirror neurons, which fire during both action execution and observation of a similar action, in the macaque monkey (Di Pellegrino et al., 1992; Gallese et al., 1996). Subsequently, electrophysiological and brain imaging studies have demonstrated a similar mirror neuron system (MNS) in humans (Fadiga et al., 1995; Grafton et al., 1996; Rizzolatti et al., 1996b; Cochin et al., 1999). The discovery of the MNS provides a possibility for explaining various social behaviors, such as imitation and intention understanding (Iacoboni et al., 1999, 2005).

Mirror neurons were originally discovered in area F5 of the monkey premotor cortex and subsequently in the rostral sector of the inferior parietal lobule (IPL) (PF/PFG) (Gallese et al., 1996; Rizzolatti et al., 1996a; Rizzolatti and Craighero, 2004; Fogassi et al., 2005). Previous studies have noted the human homolog of area F5 contains the premotor cortex [PMC: Brodmann area (BA) 6] and the inferior frontal gyrus (IFG: BA44/45), and the most likely PF/PFG in humans is located in the rostral part of the IPL (BA40) (Buccino et al., 2001; Rizzolatti and Craighero, 2004; Filimon et al., 2007; Kilner et al., 2009). In addition to being observed in homologous regions in monkeys, the superior parietal lobule (SPL: BA7) was observed in early studies of humans (Parsons et al., 1995; Buccino et al., 2001; Filimon et al., 2007; Gazzola and Keysers, 2009; Molenberghs et al., 2010). The areas mentioned above are the most reported areas in human MNS studies. Molenberghs et al. (2012) reviewed 125 fMRI studies of the MNS and concluded that BA40, followed by BA6, BA7, BA44, and BA45, was the areas reported by the largest number of studies. Accordingly, the PMC (BA6), IFG (BA44/45), SPL (BA7), and rostral IPL (BA40) are considered the dominant components of the MNS in humans (Buccino et al., 2001; Pokorny et al., 2015). Moreover, an increasing number of neuroimaging studies have suggested that these four regions play an irreplaceable role in the process of social interaction. For example, Iacoboni et al. (1999) found that the IFG and SPL were activated when subjects imitated a finger movement, suggesting that these two regions of the MNS may have an effect on human imitation. Studies have also shown that the PMC is involved in understanding the intention of others (Ciaramidaro et al., 2014) and that the rostral IPL is related to joint action (Egetemeir et al., 2011).

Functional near-infrared spectroscopy (fNIRS) is a promising, non-invasive neuroimaging approach. It is portable, cost-effective, highly flexible, and robust against head and body motions (Cui et al., 2012; Nozawa et al., 2016). This approach also represents a good compromise between temporal and spatial resolution. Compared to fMRI, fNIRS is more appropriate for exploring a wider range of experimental paradigms, especially in more naturalistic environments (Cui et al., 2012; Brucker et al., 2015; Pinti et al., 2015), and to date, a number of successful samples have been reported. For instance, Hirsch et al. (2017) established a natural interactive condition and used fNIRS to measure the brain activity of two people at the same time to explore the neural effects of eye-to-eye contact. In another study, fNIRS was applied to simultaneously record the neural signals in pairs of subjects while they played poker against each other, and the results revealed the neural mechanism of face-to-face competitive behaviors (Piva et al., 2017). Additionally, a study simultaneously scanned the neural activity of nine participants using fNIRS to investigate the neural basis of group cooperative behavior (Duan et al., 2015). Because of its high ecological validity, fNIRS has become a potentially ideal tool for exploring the MNS, which correlates with social interaction.

Considering the limited spatial coverage and spatial resolution, as well as the limited deep imaging capacity (Vanderwert and Nelson, 2014; Xiao et al., 2017), it is fundamental and indispensable to investigate the feasibility of fNIRS to detect the MNS. However, few studies have examined the feasibility. Based on the original definition of the MNS, Koehler et al. (2012) first used fNIRS to observe the activity of the MNS with a complex, everyday task. This study provided preliminary evidence for the feasibility of fNIRS to investigate the MNS (Koehler et al., 2012). However, in Koehler’s work, only posterior part of the IPL (angular gyrus) was determined to be involved in both action execution and observation. None of the four regions which were most reported in previous studies were found, which implies that fNIRS has a very limited capacity to investigate the MNS.

The purpose of our study is to investigate whether the MNS activity, including four dominant regions (the PMC, IFG, SPL, and rostral IPL), can be detected by using fNIRS. Based on the original definition of the MNS, the logic is to demonstrate that these regions are activated during both action execution and action observation. In the present study, we revised Koehler’s task and probe configuration to make it optimal for fNIRS to detect the MNS. Adopting the modified task and probe configuration, we proved the feasibility of fNIRS to investigate the MNS, using a traditional channel-based group analysis and also a ROI-based group analysis. In addition, a previous study found that neural synchronization was different between opposite gender dyads and same gender dyads in a social task (Cheng et al., 2015), and in everyday life, gender effect also exists. So, we explored whether this effect would exist in our study.

## Materials and Methods

### Subjects

We recruited 30 healthy volunteers (15 males and 15 females, mean age = 23 years, age range = 18–27 years) to participate in this study. All participants were right-handed according to the Edinburgh Handedness Questionnaire (Oldfield, 1971) and had normal or corrected-to-normal vision. No participants reported suffering from neurological or psychiatric disorders, and all the participants provided written informed consent before the experiment. This study was approved by the ethics committee of the State Key Laboratory of Cognitive Neuroscience and Learning at Beijing Normal University.

### Experimental Design

We adopted a table-setting task. The subject and the experimenter (actress) sat face to face at a table. They each had two placemats, one on their right-hand side and the other in front of them (center of the table). Each one also had a set of tableware consisting of five objects: a plate, a fork, a knife, a soup spoon, and a dessert spoon. The original positions of the tableware items were on their right-hand side. A monitor placed at a 45° angle in front of the subject presented cues.

Based on the original definition of the MNS, each subject was asked to complete two experimental conditions: an action execution and an action observation. In the execution condition, the subjects had to move the five tableware items to their designated positions with their right hand in 15 s. In Block 1, each subject moved five tableware items from the placemat on the right of the subject to another placemat in front of the subject, and in Block 2, each subject returned the tableware items to their original positions. Block 3 was the same as Block 1 and Block 4 was the same as Block 2. The order of placement was fixed: plate, fork, knife, soup spoon, and dessert spoon. The subjects were told to imagine as preparing a meal in daily life and to execute every action at a normal, natural speed. The only difference between the observation condition and the execution condition was that all the actions were completed by the actress in the former condition. In the observation condition, the subjects were instructed to watch the actions of the actress carefully and remain still.

**FIGURE 1:**
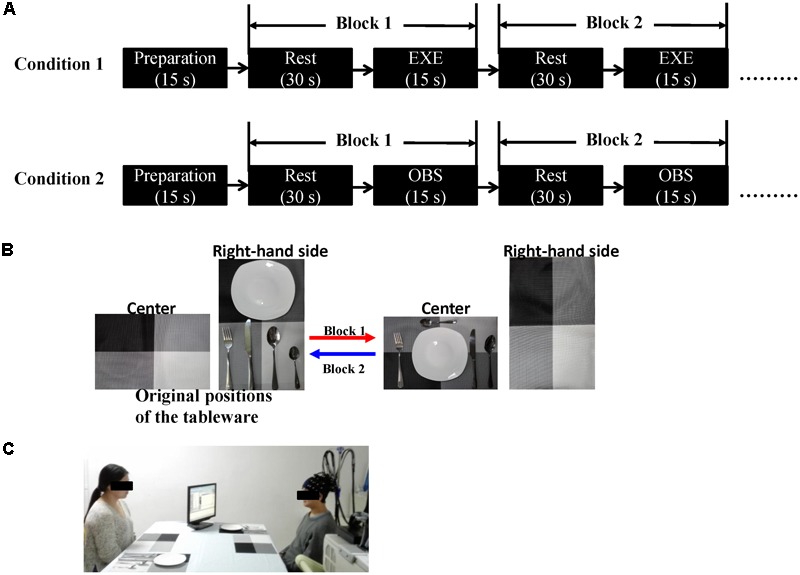
**(A)** Schematic representation of the experimental procedure. Each condition consisted of eight blocks. The initial 15 s served as preparation time so that the subjects were in a better experimental state. The order of the two conditions was counterbalanced across the subjects. **(B)** An example of the block design. In Block 1, the subject (in execution condition) or the actress (in observation condition) moved the five tableware items from the right-hand side to the center (red arrow). In Block 2, the five tableware items were returned to the original positions, the process from the center to the right-hand side (blue arrow). **(C)** Experimental scene (written informed consent was obtained from the two participants for the publication of this image). EXE, execution condition; OBS, observation condition.

Each condition consisted of eight blocks, and every block contained a 15-s task and a 30-s rest period. In the execution condition, the subjects were instructed to take a glimpse at the monitor when the cue words appeared. Upon seeing “execute,” the subjects began to execute the tasks according to the instructions and upon seeing “rest,” the subjects looked at the cross in the center of the table and avoided any actions. In the observation condition, the monitor was turned toward the actress, and the cues were the same as those in the execution condition. The subjects appropriately responded according to the actress’s actions. When the actress executed the actions, the subjects watched carefully, and when the actress stopped, the subjects rested. The order of the two conditions was counterbalanced across the subjects. Additionally, all the subjects spent some time practicing before the formal experiment to ensure that they were familiar with the rules and could adequately complete the entire task. **Figure 1** depicts the experimental design.

### fNIRS Measurement

In this study, the changes in oxyhemoglobin (HbO) and deoxyhemoglobin (HbR) concentration in each channel were measured by an optical system (LABNIRS, Shimadzu Co., Japan). This system utilizes a three-wavelength technology at 780, 805, and 830 nm.

**FIGURE 2:**
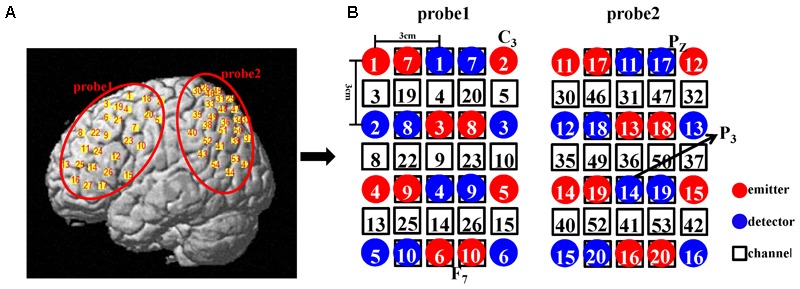
**(A)** Illustration of all the channel positions of the brain. **(B)** Schematic representation of all the optodes and channels used in our study. In the double density configuration, some emitters and detectors (e.g., emitter 7 and detector 7) were added to the original standard probe set in the same imaging area such that the distance between the two adjacent channels was 1.5 cm (e.g., channel 3 and channel 19).

We employed a double density probe configuration that was mixed by two standard probe sets (a 4 × 3 probe set and a 4 × 2 probe set). Two double density probe sets (each comprising 10 emitters, 10 detectors, and 27 channels) were attached to a regular swimming cap that was placed on the head of each subject in an identical manner (**Figure 2**). The probe sets were positioned on the left hemisphere only because the left hemisphere is dominant when the subjects perform a right-handed action (Filimon et al., 2007; Egetemeir et al., 2011). These two probe sets mostly covered the mirror-related regions which are located in the left frontal and parietal cortices in accordance with the international 10–20 system (Jasper, 1958). The sampling period was 33 ms.

### Spatial Localization

To acquire anatomical brain information, we recorded the optode and channel positions on the scalp of each subject using a 3D magnetic digitizer after he/she completed the tasks. After the experiment, each subject was allocated a location file that contained the scalp positions of all the optodes and channels.

The location file was analyzed via a probabilistic estimation process using NIRS_SPM. Each subject could provide his own anatomical labels based on various atlases for any given channel. In MNS studies, many researchers used the Brodmann area to define the mirror-related region (e.g., defining the rostral IPL as BA40). To allow comparison of our results with previous findings, the Brodmann atlas was utilized in our study. If one channel is located in areas A, B, and C, the Brodmann atlas in the NIRS_SPM toolbox produces a list (**Table 1**) for the corresponding Brodmann area and the probability of finding the scalp position in these areas. To facilitate the subsequent analysis, we used the Brodmann area possessing maximum probability as this channel’s location (Sai et al., 2014). Eventually, every subject received a location map in which every channel had only one identity and one corresponding probability. This map was the final location result.

**Table 1:** Representative example of channel location list.

Channel	Anatomical label from the Brodmann atlas	Probability
8	BA9-Dorsolateral prefrontal cortex	0.6093
	BA46-Dorsolateral prefrontal cortex	0.3907
9	BA9-Dorsolateral prefrontal cortex	0.04059
	BA44-pars opercularis, part of Broca’s area	0.76015
	BA45-pars triangularis, part of Broca’s area	0.19926

*This list was produced by the Brodmann atlas in the NIRS_SPM toolbox. The red box circles the Brodmann area with maximum probability, which is also the final location of each channel.*

### fNIRS Data Preprocessing

The fNIRS data were preprocessed and analyzed with NIRS_SPM and Matlab 2012a. To obtain a relatively stable signal, the initial 15 s (preparation time) was removed, leaving 390 s for each condition, which was down sampled to 10 Hz (Duan et al., 2015; Jiang et al., 2015). In order to remove global effects, which may be caused by blood pressure, respiration, and blood flow variation, independent component analysis (ICA) for fNIRS signals was performed (Kohno et al., 2007). Global components separated by ICA were removed. Then, we applied a second-order detrend, a discrete cosine transform-based high-pass filter (0.0078 Hz) and a low-pass filter based on the hemodynamic response function (HRF) (Huang et al., 2017).

### Traditional Channel-Based Group Analysis

First, at the individual level, the general linear model (GLM) was used to detect the hemodynamic response of HbO in all 54 channels under the two conditions, and for each subject, channel, and condition, a beta-value was generated. Second, at the group level, one-sided, one-sample *t*-tests (Koehler et al., 2012) were performed based on the individual-level beta-values to find the activated channels in both conditions (*p* < 0.01, FDR-corrected). The reason why we chose one-sided *t*-test was because we had a hypothesis about the direction of the effect, that was, the task period yielded an increased hemodynamic response compared with the rest period in both conditions.

### ROI-Based Group Analysis

**FIGURE 3:**
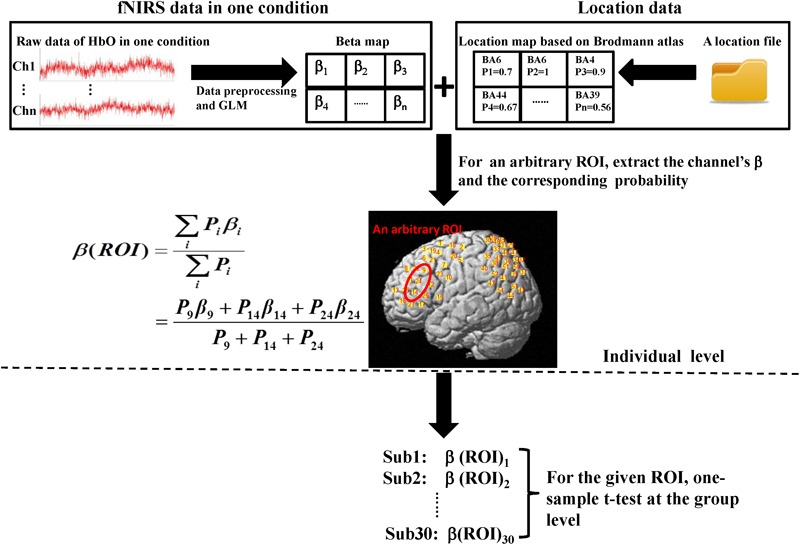
Flow diagram of ROI-based group analysis. This entire process was performed for HbO signals. Here, we adopted two experimental conditions (execution and observation) and set four ROIs including the PMC, IFG, SPL, and the rostral IPL. Therefore, the final result would get eight *t*-values at the group level. The steps shown above the dotted line are individual level analyses, which were repeated for every subject. Then, 30 weighted β for the same condition and the same ROI were tested at the group level (step below the dotted line). In both the β map and the location map shown above, each grid represents a channel and the labels of the two maps are corresponding. In the location map, every channel had only one identity according to the maximum probability.

Oxyhemoglobin changes were analyzed in three steps. The first step was the same as channel-based group analysis. According to the GLM, two beta maps (for two conditions) that included all the channels for each subject were generated. Second, for a given ROI, we extracted the beta-values of the channels which were located in the ROI based on the location map of each subject and weighted the contribution of each channel to the ROI according to its probability (Okamoto et al., 2009). Thus, for each ROI, a final beta, which was a weighted mean, was determined for every subject. Finally, at the group level, one-sided, one-sample *t*-tests were used. All analysis steps were performed for both conditions. The entire data analysis framework is presented in **Figure 3**.

### Gender Effect

The actress was fixed and half of the subjects were male and the other half were female, thus constituting both same gender pairs and opposite gender pairs. For each condition and for each mirror-related channel or ROI, a two-sided, two-sample *t*-test was performed to explore the differences between genders. This effect was analyzed only for the HbO signal.

## Results

### Activation When Using the Traditional Channel-Based Group Analysis

We first analyzed channel by channel to explore whether the activated channels in execution condition are also activated in observation condition. The results showed that 18 channels were significantly activated in both conditions (*p* < 0.01, FDR-corrected).

**FIGURE 4:**
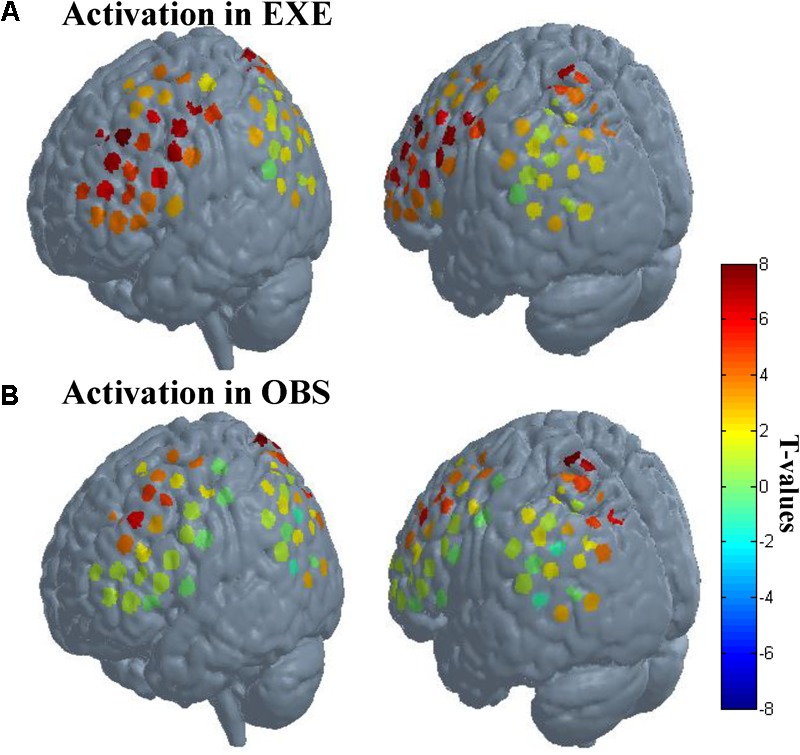
Activation when using the traditional channel-based group analysis. **(A)** Activation in execution condition. **(B)** Activation in observation condition. The probe sets were projected on the brain template-Colin 27. The left images in **(A)** and **(B)** mainly represent probe 1, and the right images mainly represent probe 2. Each circle represents one channel. The assorted colors represent the *t*-values at group level. EXE, execution condition; OBS, observation condition.

As described in the spatial localization section, each subject was assigned a location map in which each channel had only one identity based on the maximum probability from the Brodmann atlas. According to the location maps of 30 subjects, we found that the anatomical location of these activated channels were mainly PMC (BA6), IFG (BA44/45), SPL (BA7), frontal eye fields (BA8), dorsolateral prefrontal cortex (BA9), and associative visual cortex (BA19). The rostral IPL (BA40) was significantly activated in execution condition (*p* < 0.01, FDR-corrected), and in observation condition, the activation reached a significant level with a liberal threshold (*p* < 0.05, uncorrected). The *t*-values of all channels in both conditions are shown in **Figure 4**.

### Activation When Using the ROI-Based Group Analysis

**FIGURE 5:**
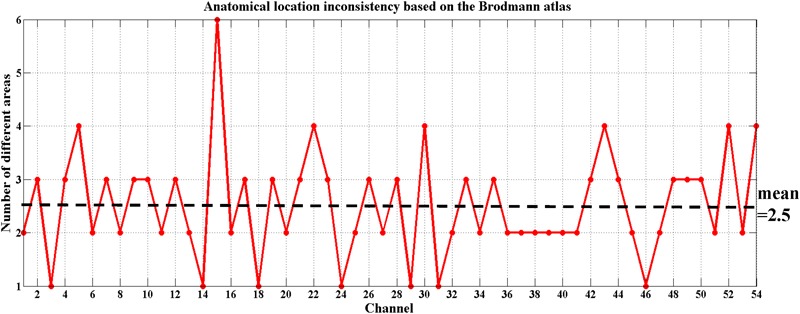
Anatomical location inconsistency based on the Brodmann atlas across 30 subjects. The *x*-axis shows 54 channels and the *y*-axis shows the number of areas expressed by 30 subjects in the same channel. Only 13% of the channels (7 out of 54) maintained consistency; i.e., only 7 of these channels were located in the same Brodmann area across all 30 subjects (number of different areas = 1). The average number of areas across 54 channels fell on 2.5.

To date, there is debate about the traditional channel-based group analysis in fNIRS studies. Channel-based group analysis assumes that the anatomical location of each channel across subjects can be considered homologous when all subjects are wearing the probes in the same manner (Tsuzuki and Dan, 2014). However, this is often not guaranteed because no two brains have the same shape or size (Dan et al., 2007), and errors are unavoidable when experimenters place the fNIRS probes on the heads of the subjects. Additionally, more channels equate to decreased placement reproducibility across subjects (Singh et al., 2005; Tsuzuki and Dan, 2014). Liu et al. (2016) also provided evidence on the inconsistency of anatomical location for the same channel across different subjects. Here, we also found the same issue when using the traditional channel-based group analysis. **Figure 5** shows the anatomical inconsistency of 54 channels across 30 subjects.

For achieving a better spatial consistency, a ROI-based group analysis is implemented (Okamoto et al., 2009; Yanagisawa et al., 2010). We set four ROIs (the PMC, IFG, SPL, and rostral IPL) and analyzed them individually to observe which ROIs are activated during both action execution and action observation. **Table 2** presents the activation results.

**Table 2:** Activation when using the ROI-based group analysis.

ROI	Definition in the	*t*-value	*p*-value	Significance
	Brodmann atlas			(FDR-corrected)
PMC	BA6	*t*_(exe)_ = 6.98	*p*_(exe)_ = 0.0000	^∗∗∗^
		*t*_(obs)_ = 3.62	*p*_(obs)_ = 0.0006	^∗∗^
IFG	BA44, BA45	*t*_(exe)_ = 6.53	*p*_(exe)_ = 0.0000	^∗∗∗^
		*t*_(obs)_ = 2.00	*p*_(obs)_ = 0.0274	^∗^
SPL	BA7	*t*_(exe)_ = 5.44	*p*_(exe)_ = 0.0000	^∗∗∗^
		*t*_(obs)_ = 7.29	*p*_(obs)_ = 0.0000	^∗∗∗^
Rostral IPL	BA40	*t*_(exe)_ = 2.24	*p*_(exe)_ = 0.0165	^∗^
		*t*_(obs)_ = 1.68	*p*_(obs)_ = 0.0519	

*The first column shows the four ROIs. The second column defines the ROI in the Brodmann atlas. PMC, premotor cortex; IFG, inferior frontal gyrus; SPL, superior parietal lobule; IPL, inferior parietal lobule; ^∗^p < 0.05; ^∗∗^p < 0.01; ^∗∗∗^p < 0.001.*

Using ROI-based group analysis, we found that three of the four ROIs, the PMC (BA6), IFG (BA44/45), and SPL (BA7), were significantly activated during both action execution and action observation. The rostral IPL was also significantly activated in execution condition, but in observation condition, the activation reached a marginal significant level (*p* = 0.0519, uncorrected). These results were consistent with the results of traditional channel-based group analysis.

To evaluate the specificity of significant activation detected by fNIRS, we selected a control region, angular gyrus (BA39). Using the ROI-based group analysis, we found that angular gyrus was activated in execution condition but not activated in observation condition (*p*_exe_ = 0.0271, *p*_obs_ = 0.4046, FDR-corrected).

**FIGURE 6:**
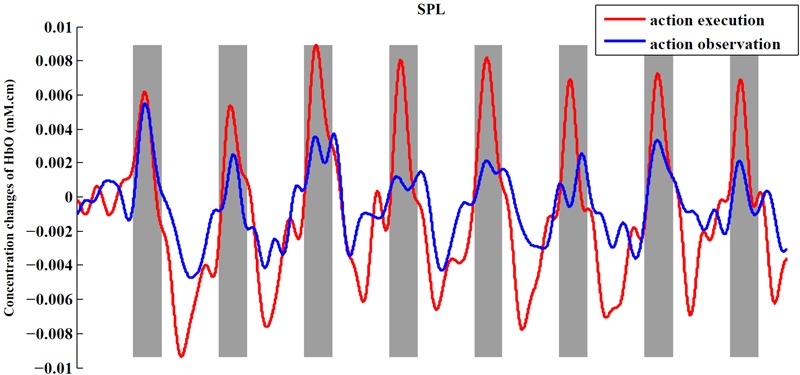
Time series for two conditions from SPL. The figure shows the averaged activities of all subjects in SPL. The process of preprocessing was similar to the section “Materials and Methods.” After preprocessing, we averaged the signals across subjects in the ROI to obtain a single time series for each condition. At last, baseline of the averaged time series was corrected according to the mean of the first rest period (30 s).

In order to observe the neural response to task intuitively, we presented time series of both experimental conditions across all subjects from an example ROI (**Figure 6**). In this figure, we can obviously see the task period (gray background) yielded an increased hemodynamic response compared with the rest period in both conditions. We also found that the magnitude of the hemodynamic response in execution condition was greater than that in observation condition.

In summary, whether using the traditional channel-based group analysis or ROI-based group analysis, we are able to find the significant activation of the MNS in both conditions, including the PMC, IFG, SPL, and rostral IPL.

### Gender Effect and Sequence Effect

We also analyzed the effect of gender on brain activation. After comparison, we found no significant difference between males and females for arbitrary experimental conditions and for arbitrary mirror-related areas. The sequence effect was analyzed in the same way, and no significant difference was observed for this effect either.

## Discussion

The present study adopted a modified task and probe configuration to evaluate the feasibility of fNIRS to investigate the MNS, whose dominant components consist of four regions, the IFG (BA44/45), adjacent PMC (BA6), SPL (BA7), and rostral IPL (BA40). The data indicated that the activity of the four regions could indeed be detected by fNIRS, using the traditional channel-based group analysis and also using the ROI-based group analysis. These findings prove that it is feasible for fNIRS to comprehensively investigate MNS activity.

Here, we detected the activity of the MNS using fNIRS where Koehler’s work did not. This reason may due to two differences between the two studies. First, in our study, the actions in observation condition were presented by a real person, while in Koehler’s study, they were presented by video. Some studies have shown that the neural response is higher when observing live actions compared to video actions (Järveläinen et al., 2001; Shimada and Hiraki, 2006). The second difference is the probe configuration. On the one hand, the probe sets of the two studies were placed on the scalp at different angles in the case of measuring similar regions. On the other hand, we used a double density probe configuration to improve image spatial resolution. The spatial resolution in our study was 1.5 cm and in Koehler’s study was 3 cm. For some narrow or smaller areas, the 3-cm spatial scale may be too rough. The feasibility of a double density configuration has been verified (Ishikawa et al., 2011).

In addition to Koehler’s work, another related study explored the brain activity during both action execution and action observation with fNIRS (Balconi and Cortesi, 2016). Subjects were asked to observe or execute an action displayed on a computer. Twenty-four channels were placed over the left central, centroparietal, parietal, and temporal areas. Contrast analysis revealed that three regions (PMC, sensorimotor cortex, and posterior parietal cortex) were associated with both action execution and action observation. We can see that except for the PMC, most of the regions in the Balconi study do not belong to the dominant components of the MNS mentioned in our study. Additionally, our experimental paradigm is different from Balconi’s. For these reasons, it is not appropriate to compare the current work with theirs.

In our study, we used two methods to demonstrate the feasibility of fNIRS to detect the MNS, and the results of the two methods were consistent. When using traditional channel-based group analysis, due to anatomical inconsistency across 30 subjects, the anatomical label at group level, for a given channel, was defined by distribution of all the subjects in different areas. For example, 80% (24/30) of the subjects showed that channel 1 was located in BA6 and 20% (6/30) of the subjects showed it was located in BA8, so we defined the channel as BA6. The significantly activated channels in the present study showed a tolerable consistency with an average of 80%. ROI-based group analysis provides an opportunity to achieve a better spatial consistency. Just as every coin has two sides, ROI-based group analysis solves the problem of inconsistency at the cost of spatial scale. Each of the two methods has its own scope of application. Maybe there will be a better method to guide the probe placement in the future to ensure that the same channel can fall on the same Brodmann area, even the same MNI coordinates of all subjects.

When presenting the time series of both conditions from an example ROI (**Figure 6**), we found that the execution condition had a greater hemodynamic response compared to the observation condition. Thus far, some studies have shown that there is a significantly greater response in mirror-related areas for action execution than for action observation (Montgomery et al., 2007; Bhat et al., 2017). So, a comparison between the action execution and observation was made in the significantly activated channels or ROIs in both conditions with two-sided paired *t*-tests. In our study, the execution condition showed a significantly greater response in the IFG, adjacent PMC, and SPL than observation condition (*p* < 0.05, uncorrected), using the traditional channel-based group analysis and also using the ROI-based group analysis. Although the difference between the two conditions in the rostral IPL did not reach a significant level, the tendency to show a greater response in execution condition was consistent with other regions.

When using ROI-based group analysis, we set a control region. The choice depends on two considerations: one is the region does not belong to MNS. The other is the region must be covered by the probe sets well. Because of the limited number of optodes and the use of double density configuration, it is difficult to cover more regions in addition to four ROIs. Although several regions are covered, for most of them, there is only one or two channels falling into and even there is no channel falling into for certain subjects. Angular gyrus (BA39) which we chose meets both of the two criteria.

The experimental paradigm we adopted closely resembles a real-life situation. The majority of previous neuroimaging studies investigating the MNS focused on functional magnetic resonance imaging (fMRI). However, the ecological validity of these studies is low, which requires participants to lie in a narrow, noisy, motionless scanner and restricts head movements to a range of 2 mm (Dinstein et al., 2010). These technical constraints make it difficult to investigate the MNS using real-life and complex actions, like the table-setting task in our study. Moreover, it is easier to extend the existing research conclusions to interpret social phenomena in daily life if the MNS is investigated in a real-life situation rather than in a restricted laboratory environment. At present, studies in real-life situations have become an advantaged field for fNIRS. It is applied to all kinds of social behaviors research in real-life situations, such as cooperation and competition (Cui et al., 2012), imitation (Holper et al., 2012), face-to-face communication (Jiang et al., 2012), decision-making (Tang et al., 2016; Zhang et al., 2017), and so on.

Here, probabilistic registration needs to be emphasized. fNIRS is a transcranial brain mapping technique, which has no anatomical brain information (Dan et al., 2007). In an fNIRS study, anatomical information is required during results presentation. When structural magnetic resonance imaging is not used, a MRI-free method, the international 10–20 system is adopted (Jasper, 1958; Herwig et al., 2003). Previous findings have demonstrated that each 10–20 landmark on the scalp corresponds to a specific cortical structure and that there is a consistent correlation between scalp positions and underlying cerebral structures across subjects (Myslobodsky et al., 1990; Okamoto et al., 2004; Xiao et al., 2017). Thus, according to the 10–20 system, attaining the approximate large-scale anatomical positions in which we are interested is achievable. However, this system is not sufficient for our study, which requires relatively accurate anatomical information, such as the Brodmann area, to identify which channels belong to the MNS. The emergence of a probabilistic registration system offers a new spatial analysis method to cope with this problem. It enables fNIRS data to be presented in standard stereotactic brain spaces. We can obtain a MNI coordinate, the Brodmann area, and automatic anatomical labeling (AAL) (Singh et al., 2005; Dan et al., 2007; Tsuzuki and Dan, 2014). Thus, probabilistic registration is essential for using fNIRS to investigate the MNS.

In our study, the PMC is defined as BA6. In the Brodmann atlas, BA6 is defined as the premotor and supplementary motor cortex. In other words, the PMC occupies part of BA6, which lies in the lateral cerebral cortex. The medial part of BA6 is the site of the supplementary motor cortex. The probe location in our study, which covered the frontal cortex, was placed mainly close to the lateral frontal cortex, and the channels located in BA6 can be attributed to the PMC. Taking a step back, some studies suggest that the supplementary motor cortex also belongs to the MNS (Gazzola and Keysers, 2009; Balconi and Cortesi, 2016).

When acquiring fNIRS data, changes in HbO and HbR concentration were measured simultaneously. However, there is controversy regarding which signal to select to analyze brain activation. In our study, we mainly focus on the HbO signal because it is often observed to have a higher amplitude than the HbR signal (Strangman et al., 2002; Yanagisawa et al., 2010). In other words, the signal-to-noise ratio of HbO is better, and this signal is more sensitive to task response (Cheng et al., 2015). We also analyzed the HbR signal and only one channel (BA7) was activated in both conditions using channel-based group analysis and also ROI-based group analysis.

Some people might question whether activation in the observation condition comes from movements of the subject. Before the experiment, all the subjects were asked to remain still during the observation condition; during the experiment, an experimenter monitored the action of subjects all the time; and after the experiment, we ensured that the subjects did not produce any explicit movements by watching their experimental videos. In our study, the subjects and the actress sat face to face, meaning the subjects observed actions with a third person view. When the experiment was completed, the subject’s self-report about perspective was submitted. In total, 28 out of 30 subjects reported that it was very easy to understand the actress’s actions by mental rotation, and only two subjects felt slightly uncomfortable, which did not affect their ability to complete the tasks. In addition, all the subjects thought that sitting face to face was closer to a real-life situation.

In the current study, among the four key regions, the response of rostral IPL (BA40) was the weakest. In the observation condition, activation of the rostral IPL reached a significant level with a liberal threshold. We tried to explore a probable reason. After analyzing the location data, we realized that because of the limited number of optodes and because we used the double density configuration, the probe sets did not cover the optimal position of the IPL. The classic mirror area is in the rostral IPL (BA40), but the probe sets mainly covered the posterior part of the IPL (BA39). There is no channel that covers the BA40 in one subject due to individual differences in brain shape and size. Thus, the activity of the mirror neurons in the rostral IPL (BA40) was most likely not well detected. Regardless of whether in the execution condition or in the observation condition, the *t*-value of the rostral IPL was the smallest among all four regions, which may support the reason that we propose. Thus, this is the limitation of our study, and perhaps additional studies can address this issue by optimizing the probe placement or increasing the effective sample size. The second limitation is the lack of a control condition. Without a control condition, it is relatively weak to interpret the activation as mirror-related responses. It is possible that the common activation may be caused by other factors presented in the two conditions, e.g., attention, visual information, and so on. In the future, a more rigorous study is needed.

## Conclusion

By employing the modified task and probe configuration described in this study, we demonstrated the feasibility of using fNIRS to investigate the activity of the MNS. This study provides a foundation for future research to explore the functional role of the MNS, which correlates with social interaction, in real-life situations using fNIRS. Furthermore, an increasing number of studies have shown that some neurological and psychiatric disorders are closely related to MNS dysfunction, including autism spectrum disorder (ASD) (Rizzolatti et al., 2009; Fakhoury, 2015) and schizophrenia (Mehta et al., 2014). Additionally, the feasibility of using fNIRS in studies involving children (Vanderwert and Nelson, 2014) and patients (Ehlis et al., 2014) has been demonstrated. Thus, our work may also support for using fNIRS in investigations of the effects of the MNS in various disorders.

## Ethics Statement

This study was carried out in accordance with the recommendations of ethics committee of the State Key Laboratory of Cognitive Neuroscience and Learning at Beijing Normal University with written informed consent from all subjects. All subjects gave written informed consent in accordance with the Declaration of Helsinki. The protocol was approved by the ethics committee of the State Key Laboratory of Cognitive Neuroscience and Learning at Beijing Normal University.

## Author Contributions

P-PS, C-ZZ, and Y-HJ designed the research. P-PS, F-LT, and Y-HJ performed the experiments. P-PS, F-LT, ZZ, and YZ analyzed the data. P-PS, F-LT, and C-ZZ drafted the work. The other authors revised the manuscript.

## Conflict of Interest Statement

The authors declare that the research was conducted in the absence of any commercial or financial relationships that could be construed as a potential conflict of interest.
