# Corrigendum: Feasibility of Functional Near-Infrared Spectroscopy (fNIRS) to Investigate the Mirror Neuron System: An Experimental Study in a Real-Life Situation

**DOI:** 10.3389/fnhum.2019.00148

**Published:** 2019-05-08

**Authors:** Pei-Pei Sun, Fu-Lun Tan, Zong Zhang, Yi-Han Jiang, Yang Zhao, Chao-Zhe Zhu

**Affiliations:** ^1^State Key Laboratory of Cognitive Neuroscience and Learning, Beijing Normal University, Beijing, China; ^2^IDG/McGovern Institute for Brain Research, Beijing Normal University, Beijing, China; ^3^Center for Collaboration and Innovation in Brain and Learning Sciences, Beijing Normal University, Beijing, China

**Keywords:** social interaction, mirror neuron system, fNIRS, real-life situation, double density, channel-based group analysis, ROI-based group analysis

In the original article, there was a mistake in Table 2 as published. The last two lines in the first two columns of the table were presented in reverse order. The corrected table appears below.

**Table 2:** Activation when using the ROI-based group analysis.

**ROI**	**Definition in the Brodmann atlas**	***t*-value**	***p*-value**	**Significance (FDR-corrected)**
PMC	BA6	*t*_(exe)_ = 6.98	*p*_(exe)_ = 0.0000	***
		*t*_(obs)_ = 3.62	*p*_(obs)_ = 0.0006	**
IFG	BA44, BA45	*t*_(exe)_ = 6.53	*p*_(exe)_ = 0.0000	***
		*t*_(obs)_ = 2.00	*p*_(obs)_ = 0.0274	*
SPL	BA7	*t*_(exe)_ = 5.44	*p*_(exe)_ = 0.0000	***
		*t*_(obs)_ = 7.29	*p*_(obs)_ = 0.0000	***
Rostral IPL	BA40	*t*_(exe)_ = 2.24	*p*_(exe)_ = 0.0165	*
		*t*_(obs)_ = 1.68	*p*_(obs)_ = 0.0519	

*The first column shows the four ROIs. The second column defines the ROI in the Brodmann atlas. PMC, premotor cortex; IFG, inferior frontal gyrus; SPL, superior parietal lobule; IPL, inferior parietal lobule*;

* *p < 0.05*;

** *p < 0.01*;

*** *p < 0.001*.

In the original article, there was also an error in the text. In one passage, there were two commas missing and one redundant word “and”. A correction has been made to the section Materials and Methods, sub-section Traditional Channel-Based Group Analysis. The corrected sentence is presented below.

“The reason why we chose one-sided *t*-test was because we had a hypothesis about the direction of the effect, that was, the task period yielded an increased hemodynamic response compared with the rest period in both conditions.”

The authors apologize for these errors and state that this does not change the scientific conclusions of the article in any way. The original article has been updated.

